# White saffron (*Curcuma mangga* Val.) attenuates diabetes and improves pancreatic β-cell regeneration in streptozotocin-induced diabetic rats

**DOI:** 10.1016/j.toxrep.2022.05.014

**Published:** 2022-05-20

**Authors:** Dwiyati Pujimulyani, Wisnu Adi Yulianto, Astuti Setyowati, Prastyo Prastyo, Sulkhan Windrayahya, Ali Maruf

**Affiliations:** a Faculty of Agroindustry, University of Mercu Buana Yogyakarta, Jl. Wates Km 10, 55753 Yogyakarta, Indonesia; b Particle and Interfacial Technology Group (PaInT), Department of Green Chemistry and Technology, Ghent University, Coupure Links 653, 9000 Ghent, Belgium; c Centre for Food and Microbial Technology, KU Leuven, Kasteelpark Arenberg 22, B-3001 Haverlee, Belgium; d Biotechnology Center, The Silesian University of Technology, Krzywoustego 8, 44-100 Gliwice, Poland; e Department of Organic Chemistry, Bioorganic Chemistry and Biotechnology, The Silesian University of Technology, Krzywoustego 4, 44-100 Gliwice, Poland

**Keywords:** Diabetes, Glucose, Insulin, Pancreatic β-cells, White saffron

## Abstract

Diabetes is a chronic disease caused by an imbalance of insulin release to the bloodstream in response to excessive glucose influx, which causes hyperglycemia. White saffron (*Curcuma mangga* Val.), an Indonesian aromatic spice, contains essential phytochemicals and has a number of potential health benefits. Here, we examined the effects of oral administration of white saffron powder (WSP): 1.5 and 4.5 g (WSP 1.5 and WSP 4.5) in streptozotocin (STZ)-induced diabetic rats. WSP was administered orally on a daily basis for one month and its antidiabetic, anti-inflammatory, and antioxidative effects were investigated by measuring the concentrations of blood glucose, insulin, interleukin 6 (IL-6), tumor necrosis factor-α (TNF-α), interleukin 8 (IL-8), malondialdehyde (MDA), and superoxide dismutase (SOD) activity. In response to high WSP intervention (WSP 4.5), treated rats showed increased insulin level and SOD activity and reduced blood glucose, IL-6, IL-8, TNF-α, and MDA levels, which were closely related to the positive control (PC) group. In addition, Hematoxylin and Eosin (H&E) staining of the pancreatic tissues showed that WSP 4.5-treated rats had significant improvement in β-cell regeneration, which taken together reflected the antidiabetic potential of *Curcuma mangga* Val.

## Highlights

• White saffron is a promising aromatic spice with antidiabetic effects.
• White saffron powder increased the insulin level and reduce the glucose level.
• White saffron powder reduced pro-inflammatory cytokines IL-6, IL-8, and TNF-α.
• White saffron powder reduced MDA level and increased SOD activity.
• White saffron powder improved β-cell regeneration of the damaged pancreas.

## Graphical Abstract

**Figure:**
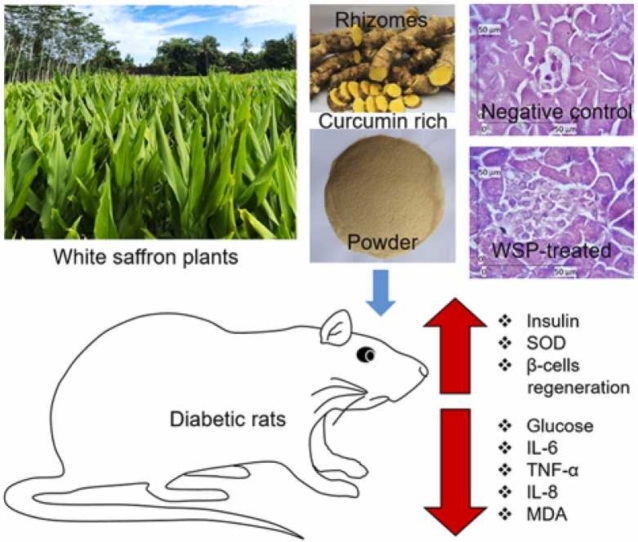

## 1 Introduction

Diabetes mellitus, commonly known as diabetes, is a chronic disease that predominantly changes the metabolism of macronutrients (e.g., carbohydrates, proteins, and fats), electrolytes, and water due to abnormal insulin secretion. The number of population worldwide suffering from diabetes was estimated around 451 million people in 2017 [1]. Notably, about 80 million of them were in Southeast Asia, and it was predicted to reach 150 million by 2045 [1]. Data from the World Health Organization (WHO) in 2016 revealed that the prevalence of diabetes among Indonesians was around 7.0% [2]. The primary risk factors, including overweight, obesity, and physical inactivity accounted for 24.4%, 5.7%, and 22.8%, respectively [2]. Other factors, such as population growth, increased numbers of elderly people, urbanization, eating patterns, and unhealthy lifestyles are believed to escalate diabetes cases in the future.

Diabetes can cause acute complications, such as vascular abnormalities (microangiopathy and macroangiopathy) [3]. The former is mainly found in diabetic retinopathy and nephropathy that can cause blindness and kidney failure [3], [4]. Meanwhile, macroangiopathy can be found in the lower limbs and blood vessels that can cause gangrene and coronary heart disease [5], [6]. Therefore, effective, affordable, and safe treatments are necessary, especially by using local materials that are easily obtained.

Recently, various types of medicinal plants have been explored to cure diabetes, such as *Mangifera indica*, *sambiloto* (*Andrographis paniculata* [Burn.f.] Ness), ngai camphor, Madagascar periwinkle, green tea, Java plum, Java tea, mahogany (*Swietenia mahagoni* Jacq), *Plectranthus esculenthus* N.E.Br, and Indonesian bay leaf (*Syzygium polyanthum* [Wight.] Walp) [7], [8], [9]. White saffron (*Curcuma mangga* Val.), a natural aromatic spice, is widely grown in Indonesia and commonly used as traditional medicine as well, which is linked to its rich content in curcuminoid [10].

Due to the increasing popularity of medicinal plants as alternative sources of treatment, many studies have been conducted to explore their potential for clinical relevance. For instance, the pharmacological characteristics of phytochemical compounds from different medicinal plants have been studied to treat different kinds of chronic diseases, such as diabetes and hypertension [11], [12]. The contained bioactive phytochemicals (e.g., phenolic compounds, flavonoids, coumarin, and terpenoid) might play a role in glucose-lowering effects [11], [12].

In our previous study, we have studied in vitro hypoglycemic activity of white saffron, which was related to its ability to modulate glucose transporter type 4 (GLUT4), a transmembrane receptor that is responsible for insulin-dependent glucose uptake [13]. At the same time, white saffron could reduce peroxisome proliferator activated receptor γ (PPAR-γ) expression in adipocytes, which is relevant to its function to regulate lipid deposition [13]. In the current study, we attempted to study in vivo antidiabetic, anti-inflammatory, and antioxidative activities of white saffron in STZ-induced diabetic rats. Low and high dosages of WSP were administered to diabetic and non-diabetic rats for a month to compare their effects. H&E staining of pancreatic tissues was also conducted to compare β-cells integrity.

## 2 Materials and Methods

### 2.1 Materials

White saffron was planted in manure-enriched soil in a 4000-meter square private garden located in Sedayu, Bantul, Yogyakarta. Ready-to-harvest white saffron (mature, aged 10 months) is characterized by its fallen leaves, bright yellow-flesh rhizomes, and mango-like smell. After harvesting, white saffron rhizomes (all parts: main rhizome, first branch, and second branch) were washed, peeled, and blanched with hot water containing 0.05% citric acid for 5 min. Then, they were cut into small pieces, dried in a cabinet dryer, and ground to obtain WSP.

### 2.2 Measurement of curcumin content in white saffron

**Fig. 1:**
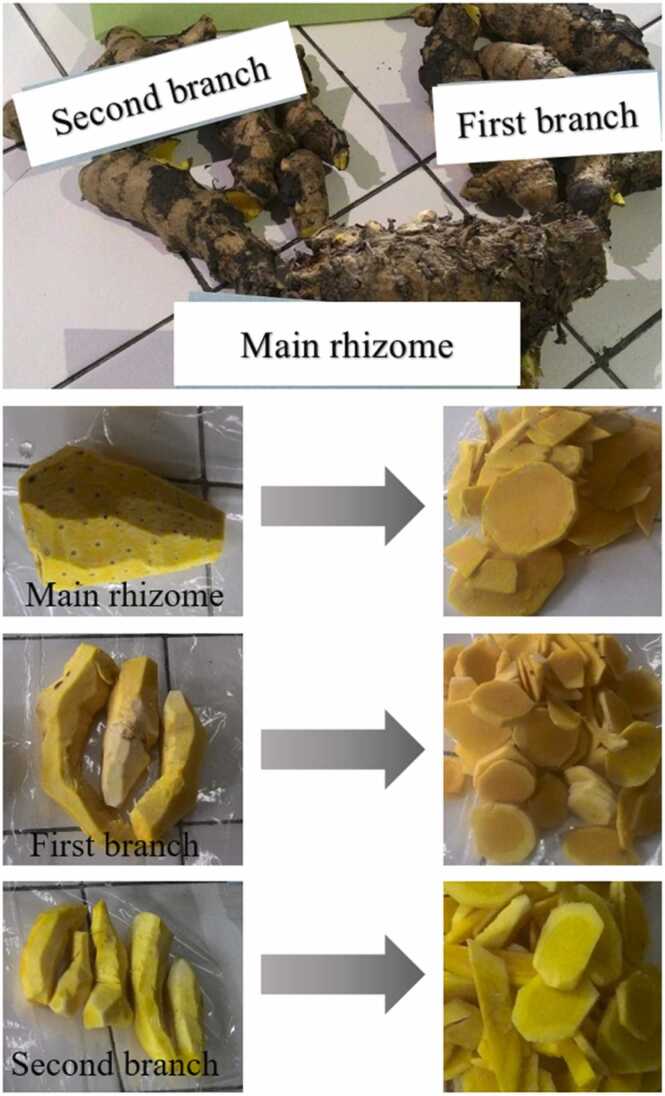
White saffron rhizome and its parts: main rhizome, first branch, and second branch (before and after peeling and slicing).

Curcumin content of white saffron was measured from different parts of the rhizomes, including the main rhizome, first branch, and second branch (Fig. 1). Initially, fresh white saffron was ground and extracted with ethanol and its total curcuminoid content was quantified spectrophotometrically at the wavelength of 425 nm. For quantifying the curcumin content, 5 µL of the filtrate was introduced into thin-layer chromatography (silica gel 60 F_254_) and put into a chamber containing chloroform:methanol mobile phase (98:2 v/v). The plate was taken and dried once it was thoroughly developed with the eluent. The absorbance of the formed spots was measured by a densitometer at 425 nm, and the curcumin content was determined from the total curcuminoid.

### 2.3 Animal study

**Fig. 2:**
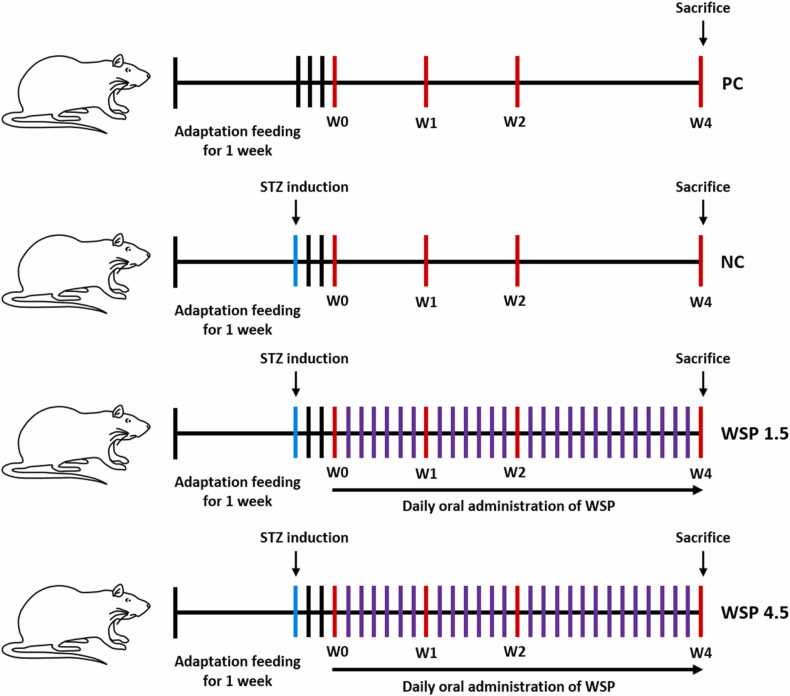
A schematic diagram of the animal study. Twenty four white rats (*Rattus norvegicus* L.) were divided into four groups: PC, NC, WSP 1.5, and WSP 4.5 (6 rats for each group). Adaptation feeding (standard diet) was given for one week, following STZ induction (blue lines) for NC, WSP 1.5, and WSP 4.5 groups. After 3 days of STZ induction, rats were measured their blood glucose levels (red lines) for confirmation of diabetes status, stated as week 0 (W0). Then, daily oral WSP was given for WSP 1.5 and WSP 4.5 groups until W4 (purple lines). At W1, W2, and W4, rats were analyzed their blood glucose, insulin, IL-6, IL-8, TNFα, and MDA levels as well as SOD activity. At the end of the study, all rats were sacrificed for obtaining their pancreatic tissues. (For interpretation of the references to colour in this figure legend, the reader is referred to the web version of this article.)

Twenty four white rats (*Rattus norvegicus* L.) (male, aged 8 weeks, bodyweight: 180–210 g) were divided into four groups, namely PC, NC, WSP 1.5, and WSP 4.5 (6 rats for each group). Adaptation feeding (standard diet) was given for one week, following STZ induction for NC, WSP 1.5, and WSP 4.5 groups by a single intraperitoneal (IP) injection (45 mg/kg) in citrate buffer (pH 4.5). Meanwhile, the PC group was normal rats without STZ induction. After 3 days of STZ induction, rats were measured their blood glucose levels for confirmation of diabetes status (higher than 250 mg/dL is considered diabetes), stated as week 0 (W0) [14], [15], [16]. WSP was given orally using a blunt microsyringe, and the study was conducted for one month (Fig. 2). To assess the antidiabetic, anti-inflammatory, and antioxidative activities of WSP in diabetic rats, the representative variables, including blood glucose and insulin, IL-6, IL-8, and TNFα, and SOD and MDA were measured at different time intervals (week 1, 2, and 4: W1, W2, and W4, respectively). The animal study was conducted ethically in the Center for Food and Nutrition Studies (PSPG UGM), Gadjah Mada University, Indonesia, under the approval of the Health Research Ethics Committee, Faculty of Health Science, Yogyakarta Respati University (UNRIYO), Yogyakarta, Indonesia (ethical clearance number: 180.3/FIKES/PL/VII/2019).

### 2.4 Red blood cells and blood plasma sample preparation

Rats were anesthetized intramuscularly with ketamine (60 mg/kg) and then their blood were withdrawn from the orbital sinus route using EDTA as the anticoagulant and the obtained blood was transferred into an eppendorf tube following centrifugation at 3000 rpm for 10 min to isolate red blood cells (RBCs) and blood plasma for further assays.

### 2.5 Determination of blood glucose level

Glucose level was quantified using the commercially available DiaSys kit (DiaSys Diagnostic System, Germany). The kit has a sensitive determination of glucose concentrations from 1 to 400 mg/dL. Glucose determination was done after enzymatic oxidation by glucose oxidase. Quinoneimine was used as the colorimetric indicator, which was generated via hydrogen peroxide-mediated oxidation of 4-aminoantipyrine and phenol (peroxidase was used as the catalyst, Trinder’s reaction). Briefly, freshly isolated blood plasma (10 µL) or standard (100 mg/dL, 10 µL) was mixed with 1.0 mL of reagent containing phosphate buffer (pH 7.5), phenol, 4-aminoantipyrine, glucose oxidase, and peroxidase. Then, the mixture was incubated at 25 ºC for 10 min, following the absorbance measurement at a 500-nm wavelength. The glucose concentration was measured by comparing the absorbance of samples with the standard, which was expressed in mg/dL.

### 2.6 Determination of insulin level

An enzyme-linked immunosorbent assay (ELISA) kit (Fine-Test, China) was employed to measure the insulin level following the given instruction. Briefly, 100 µL of standard curve solutions, known diluted blood plasma samples, and blank (dilution buffer) were placed into a 96-well plate, sealed, and incubated at 37 ºC for 1.5 h. Then, the solution was discarded and washed twice with washing buffer before adding 100 µL of biotin-labeled antibody. The plate was then incubated again for another 1 h. After incubation finished, solutions were removed and washed thrice with washing buffer (1–2 min gentle shaking for each wash). Immediately, 100 µL of horseradish peroxidase (HRP)-streptavidin conjugate (SABC) solution was added into each well following incubation at 37 ºC for 30 min. Then, solutions were again removed and washed five times with washing buffer. To visualize HRP enzymatic reaction, each well was added with 3,3′,5,5′-tetramethylbenzidine (TMB) substrates (90 µL) following the final incubation for 10–20 min in dark conditions. To stop the reaction, stop solution (50 µL) was added into each well, yielding a yellow color. The plate was then placed into a microplate reader to measure its absorbance at 450 nm and then the insulin concentration was calculated based on the standard curve.

### 2.7 Determination of IL-6, TNFα, and IL-8 levels

IL-6, TNFα, and IL-8 levels were also measured using an ELISA kit (Fine-Test, China), following the same procedure as the kit for measuring insulin level. However, every kit used a different standard with different ranges of a standard curve (the detection sensitivity is different). The procedure of measurement was as follows. Briefly, 100 µL of standard curve solutions, known diluted blood plasma samples, and blank (dilution buffer) were placed into a 96-well plate, sealed, and incubated at 37 ºC for 1.5 h. Then, the solution was discarded and washed twice with washing buffer before adding 100 µL of biotin-labeled antibody. The plate was then incubated again for another 1 h. After incubation finished, solutions were removed and washed thrice with washing buffer (1–2 min gentle shaking for each wash). Immediately, 100 µL of SABC solution was added into each well following incubation at 37 ºC for 30 min. Then, solutions were again removed and washed five times with washing buffer. To visualize HRP enzymatic reaction, each well was added with 90 µL of TMB substrates following the last incubation for 10–20 min in dark conditions. To stop the reaction, stop solution (50 µL) was added into each well, yielding a yellow color. The plate was then placed into a microplate reader to measure its absorbance at 450 nm and then the IL-6, TNFα, and IL-8 concentrations were calculated based on the standard curve.

### 2.8 Determination of SOD activity

SOD activity was measured using the SOD activity kit (GenWay Biotech Inc., USA), following the procedure from the manufacturer. Briefly, 500 µL of isolated RBCs were diluted into 2.5 mL (5 ×) using cold distilled water, mixed, and centrifuged at 10000g for 10 min to pellet the RBC membranes. Then, the obtained supernatant was collected and diluted 100 × prior to SOD activity assay. To conduct the assay, three different blanks were prepared for each sample in different wells in a 96-well plate. Firstly, blank 1 consisted of distilled water, water-soluble tetrazolium salts (WST) working solution, and enzyme working solution (20, 200, and 20 µL, respectively). Secondly, blank 2 consisted of sample solution, WST working solution, and dilution buffer (20, 200, and 20 µL, respectively). Thirdly, blank 3 consisted of distilled water, WST working solution, and dilution buffer (20, 200, and 20 µL, respectively). Finally, each sample solution (20 µL) was mixed with WST working solution (200 µL) and enzyme working solution (20 µL). Then, the plate was incubated at 37 ºC for 20 min, following an immediate measurement of absorbance in a microplate reader (450 nm wavelength). SOD activity was expressed as % inhibition rate, by using this equation:

$$\mathrm{SOD activity}(\%\mathrm{inhibition rate})\;=\frac{\left(\mathrm{Abs}\;\mathrm{blank}\;1-\mathrm{Abs}\;\mathrm{blank}\;3\right)-(\mathrm{Abs}\;\mathrm{sample}-\mathrm{Abs}\;\mathrm{blank}\;2)}{\left(\mathrm{Abs}\;\mathrm{blank}\;1-\mathrm{Abs}\;\mathrm{blank}\;3\right)}\;\times 100$$

### 2.9 Determination of MDA level

A thiobarbituric acid reactive substance (TBARS) method was employed to estimate the MDA level. Briefly, a solution of phosphoric acid:blood plasma (15:1 v/v, 800 µL) was freshly prepared in 15-mL polypropylene tube and mixed with thiobarbituric acid solution (250 µL, 40 mM). The obtained mixture was diluted with distilled water (450 µL), gently vortexed, and heated for 1 h (the lid was closed tightly during heating). Then the solution was cooled down in an ice bath, mixed gently, and applied to a prewashed Sep-Pak C18 column. Methanol (4 mL) was used to elute TBARS from the column. Finally, the MDA level was measured from a generated standard curve of tetraethoxypropane (TEP) in a spectrophotometric method (wavelength: 532 nm), where the level of lipid peroxides was indicated as nmol of MDA.

### 2.10 H&E staining

The euthanasia procedure was conducted by a single injection of ketamine solution (100 mg/kg). Then, rats were dissected and harvested their pancreatic tissues, following fixation in Bouin’s solution. After fixation, the tissues were dehydrated, embedded in paraffin, and sectioned at 5 µm. The selected tissue sections were then stained with H&E for comparing the histological characteristics between samples, especially islets of Langerhans and β-cells integrity.

### 2.11 Statistical analysis

The obtained data were expressed as mean ± standard deviation (SD) of six repetitions (*n=* 6). Statistical analysis was conducted to measure any significant differences using one-way analysis of variance (ANOVA, first stage) and Duncan's Multiple Range Test (DMRT, post hoc test) with a 95% confidence interval (*p* < 0.05). In addition, to determine a significant difference between two selected groups, unpaired two-tail Student's t-test with significant levels set to ^ns^*p* > 0.05, * *p* < 0.005, * * *p* < 0.01 * ** *p* < 0.001, and * ** * *p* < 0.0001 was included.

## 3 Results and Discussions

### 3.1 Curcumin content in white saffron

*Curcuma mangga* Val. contains many bioactive compounds including curcumin, gallic acid (GA), catechin (CA), epicatechin (EP), epigallocatechin (EPG), epigallocatechin gallate (EPGG), and gallocatechin gallate (GG). In our previous study, we have characterized the GA, CA, EP, EPG, EPGG, and GG contents in fresh *Curcuma mangga* Val. extract, namely 12.4, 13.4, 44.2, 11.3, 3.7, and 15.9 mg/100 g dried extract, respectively [17]. In the present study, we measured the curcumin content of *Curcuma mangga* Val. from different parts of the rhizomes. The main rhizome had more than double the curcumin content of the first and second branches, which were 88.6 vs 37.5 and 31.4 mg/100 g dried extract, respectively (Table 1).

**Table 1:** Curcumin contents of fresh *Curcuma mangga* Val. extract from different parts of the rhizomes.

Rhizome parts	Curcumin content (mg/100 g dried extract)
Main rhizome	88.6
First branch	37.5
Second branch	31.4

### 3.2 White saffron maintains rats bodyweight

**Fig. 3:**
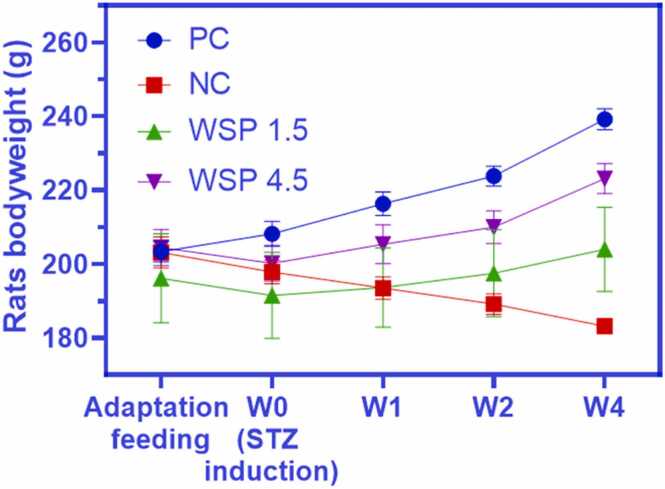
Measurement of rats bodyweight from the beginning of study until W4 of WSP treatment. Data are presented as mean ± SD (*n* = 6).

As seen from Fig. 3, the bodyweight of diabetic rats without WSP intervention gradually declined from 203.2 to 183.2 g. Meanwhile, the PC group showed increased bodyweight from 203.3 to 239.2 g. After treatment with WSP 1.5 and 4.5, the bodyweight of rats indicated a steady increase, in which WSP 4.5 showed a significant increase of the bodyweight at the end of study. Similar results were reported on STZ-induced diabetic rats [18]. STZ caused a significant reduction in the bodyweight of albino rats as observed after 2, 4, 6, and 8 weeks post-induction [18].

### 3.3 White saffron reduces glucose level and improves insulin level

**Fig. 4:**
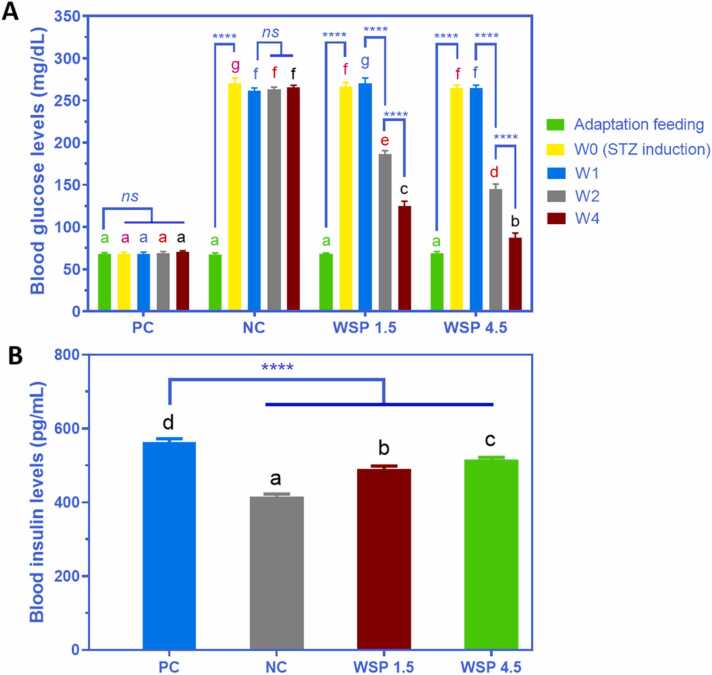
(A) Blood glucose levels of diabetic rats after treatment with WSP 1.5 and WSP 4.5 for one week (W1), two weeks (W2), and four weeks (W4). Adaptation feeding was given before STZ induction and confirmation of diabetes status, stated as week 0 (W0). (B) Blood insulin levels of diabetic rats after treatment with WSP 1.5 and WSP 4.5 for four weeks. PC: non-diabetic rats, NC: diabetic rats. Data are presented as mean ± SD (*n* = 6). Different notations with the same color (green, pink, blue, red, or black) indicate a significant difference among groups. (For interpretation of the references to colour in this figure legend, the reader is referred to the web version of this article.)

In general, diabetic rats had blood glucose levels nearly four times higher than non-diabetic rats, as shown in Fig. 4A, while the figures for WSP 1.5 and 4.5 exhibited a visible drop in blood glucose levels from W1 to W4. In the WSP 1.5 group, blood glucose level decreased from 270.39 mg/dL (W1) to 186.33 and 125.10 mg/dL (W2 and W4, respectively). Similarly, in the WSP 4.5 group, blood glucose level decreased from 264.77 mg/dL (W1) to 144.54 and 87.66 mg/dL (W2 and W4, respectively). These results indicated that the reduction in the glucose level was dose- and time-dependent with the optimal treatment was WSP 4.5, in which circulating glucose was almost reaching the normal level after one month of treatment.

According to Widowati (2008), the mechanism of action of various plants as antidiabetic agents can be divided into three parts [19]. (1) They can agglomerate intestinal-mucosa membrane protein and build a protective layer that can inhibit glucose intake and normalize blood glucose rate. (2) They can accelerate blood circulation and kidney filtration and excretion that enhance urine production-mediated glucose excretion through the kidneys so that the glucose level in the blood will decrease. (3) They can accelerate glucose excretion through increased metabolism or fat storage mediated by the pancreas to produce insulin [19].

White saffron contains an abundance of phenolic compounds [10], [17]. Phenolic compounds such as flavonoids, phenols, flavonols, and proanthocyanidins in plants have potential as antioxidants and antidiabetic agents [20]. Flavonoids that are beneficial to diabetic patients include interradiain, quercetin, rutin, diosmin, luteolin, CA, and cinnamic acid [20], [21]. Flavonoids appear as aglycones and glycosylated and methylated derivatives [22]. Flavonoid compounds are believed to have antidiabetic effects due to their antioxidant properties that can protect our bodies against free radicals and other oxidizers. A recent study showed that *Cistus laurifolius* L. contained GA, CA, EP like *Curcuma mangga* Val. and other bioactive compounds (e.g., rutin, *p*-coumaric acid, and resveratrol) and its treatment (250 mg/kg *C. laurifolius* L. extract) in STZ-induced diabetic rats could reduce blood glucose level from 355 to 288 mg/dL and increase insulin level from 9.5 to 14.8 μU/mL [23].

On the other hand, Fig. 4B shows that the insulin level was the lowest in diabetic rats, while rats given WSP 1.5 and 4.5 for four weeks showed relatively high insulin levels close to the normal rats, which were 491.02 and 516.52 pg/mL, respectively. White saffron contains curcumin, which acts as antioxidant. A recent study showed that curcumin could maintain β-cell function in vivo by inhibiting phosphodiesterase activity [24]. As a result, the production of insulin could be regulated due to the normal function of β-cells.

### 3.4 White saffron reduces IL-6, TNF-α, and IL-8 levels

**Fig. 5:**
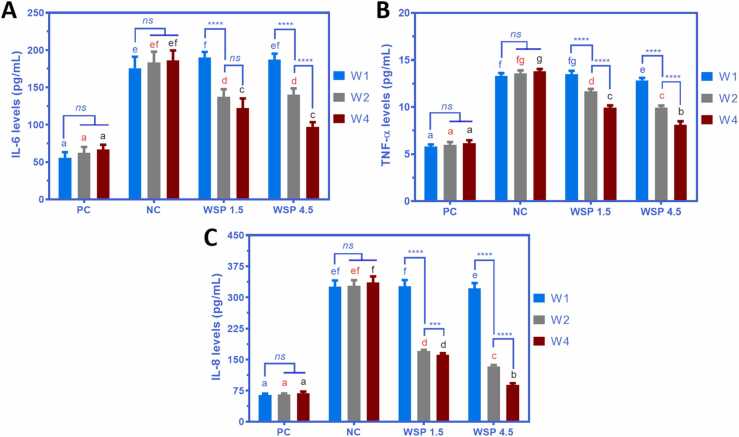
(A) IL-6, (B) TNF-α, and (C) IL-8 levels of diabetic rats after treatment with, WSP 1.5 and WSP 4.5 for one week (W1), two weeks (W2), and four weeks (W4). PC: non-diabetic rats, NC: diabetic rats. Data are presented as mean ± SD (*n* = 6). Different notations with the same color (blue, red, or black) indicate a significant difference among groups. (For interpretation of the references to colour in this figure legend, the reader is referred to the web version of this article.)

The anti-inflammatory effects of WSP against diabetes are shown in Fig. 5A-C. It is clear that diabetic rats had much higher IL-6, TNF-α, and IL-8 levels than the normal rats. Meanwhile, diabetic rats treated with WSP 1.5 and 4.5 showed a significant decrease in IL-6, TNF-α, and IL-8 levels from W1 to W4. In the WSP 1.5 group, IL-6, TNF-α, and IL-8 levels of diabetic rats decreased from 190.25, 13.49, and 326.68 pg/mL (W1) to 137.17, 11.66, 170.48 pg/mL (W2) and 122.10, 9.94, and 161.68 pg/mL (W4), respectively. Similarly, in the WSP 4.5 group, IL-6, TNF-α, and IL-8 levels of diabetic rats dropped from 187.42, 12.84, and 322.10 pg/mL (W1) to 140.10, 9.94, and 134.02 pg/mL (W2) and 97.39, 8.11, and 89.09 pg/mL (W4), respectively.

The phenolic and flavonoid compounds from white saffron might play a key role in reducing those pro-inflammatory cytokines [25]. IL-6 is mainly secreted by white blood cells and its overproduction might induce insulin resistance [26]. Meanwhile, TNF-α, one of the critical inflammatory cytokines, is secreted by macrophages/monocytes during severe inflammation and plays a primary role in various cellular signaling pathways [27], [28]. The high level of TNF-α in the bloodstream is associated with multiple chronic diseases, including cardiovascular diseases, inflammatory bowel disease, and diabetes [27], [28]. On the other hand, IL-8 is a chemokine that directly contributes to macrophage infiltration and activation in adipose tissue and has been studied to get involved in type 2 diabetes and atherosclerosis [29]. WSP intervention for one month could reduce the IL-6, TNF-α, and IL-8 levels in diabetic rats.

### 3.5 White saffron improves SOD activity and reduces MDA level

**Fig. 6:**
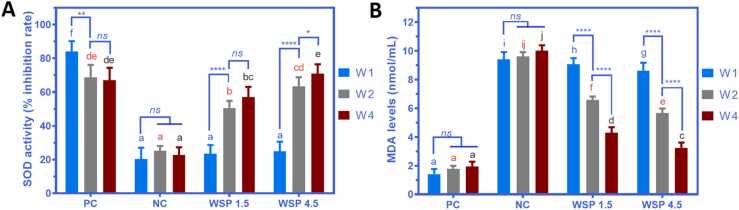
(A) SOD activity and (B) MDA level of diabetic rats after treatment with WSP 1.5 and WSP 4.5 for one week (W1), two weeks (W2), and four weeks (W4). PC: non-diabetic rats, NC: diabetic rats. Data are presented as mean ± SD (*n* = 6). Different notations with the same color (blue, red, or black) indicate a significant difference among groups. (For interpretation of the references to colour in this figure legend, the reader is referred to the web version of this article.)

According to the data in Fig. 6 A,B, it is clear that, as increased dose and prolonged time of WSP treatment, the SOD activity of diabetic rats increased considerably, while the MDA level of diabetic rats declined significantly compared to the NC group. In the WSP 1.5 group, the SOD activity of diabetic rats increased from 23.53% (W1) to 50.63% and 57.05% (W2 and W4, respectively), while the MDA level decreased from 9.08 nmol/mL (W1) to 6.59 and 4.28 nmol/mL (W2 and W4, respectively). Similarly, in the WSP 4.5 group, the SOD activity of diabetic rats increased from 24.84% (W1) to 63.52% and 70.83% (W2 and W4, respectively), while the MDA level decreased from 8.62 nmol/mL (W1) to 5.66 and 3.25 nmol/mL (W2 and W4, respectively). After treatment with WSP 1.5 and 4.5 for one month, both SOD activity and MDA level of diabetic rats were closely related to the PC group, while the NC group showed relatively low SOD activity and high MDA level, as seen in Fig. 6.

White saffron antioxidants in the form of curcumin and polyphenols could increase SOD levels as the forefront of defense against free radicals [10], [30]. Recently, Pujimulyani et al. (2020) [31] have studied an oral administration of WSP to the oxidized peanut oil-treated wistar rats. The results showed that WSP treatment could increase the SOD and vitamin E levels and reduce the MDA levels [31]. MDA is the final product of lipid oxidation. High MDA levels are influenced by lipid peroxide levels, which also indirectly indicate a high amount of free radicals [32]. In the previous study, white saffron extract and fractions showed the ability to capture free radicals, such as nitric oxide (NO) and hydrogen peroxide (H_2_O_2_) [25].

### 3.6 White saffron maintains β-cells integrity

Pancreatic β-cells play a primary role in producing and releasing insulin in a strictly controlled manner in order to keep the circulation of glucose at a normal range [33]. It is crucial to preserve β-cell structure and function and prevent damages that can cause cell death-induced disrupted cell integrity. According to Anděl et al. (2014) [34], various factors could damage or destroy β-cells, including metabolic factors (e.g., hyperglycemia, lipotoxicity, and reactive oxygen species), pharmacological factors (e.g., antimicrobial medication and antidepressants), cystic fibrosis (e.g., infections, inflammation, and autoimmunity), environmental toxic factors (e.g., STZ and rat poison), impaired insulin secretion, exocrine disorders, and viruses [34].

**Fig. 7:**
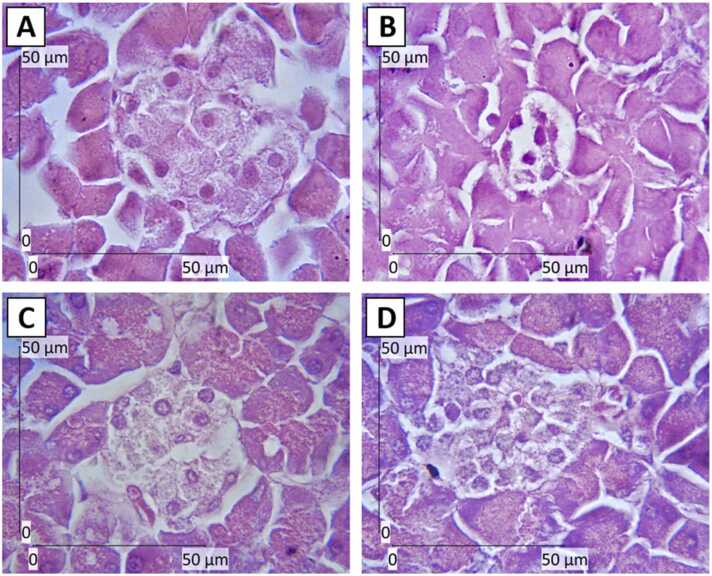
H&E staining of pancreatic tissues, showing the islets of Langerhans and β-cell integrity after treatment with (C) WSP 1.5 and (D) WSP 4.5 for four weeks. (A) PC: non-diabetic rats, (B) NC: diabetic rats. Scale bar: 50 µm.

From Fig. 7A-D, it is clear that the NC group showed disrupted β-cells structure and integrity compared to the PC group, which was caused by diabetes. β-cells and the islets of Langerhans in the PC group were in normal condition. Meanwhile, WSP 1.5 and WSP 4.5 groups indicated a significant improvement in β-cells regeneration compared to the NC group. There are three different types of impaired functions on the destruction of β-cells, namely decreased sensitivity to glucose, impaired insulin secretion (biphasic profile and pulsatility), and decreased β-cell mass-induced uncontrolled glucose homeostasis [35], [36].

Natural compounds in various forms, such as berberine, curcumin, and mangiferin as well as extracts of medicinal plants have been found to have protective and regenerative effects on β-cells [36]. For instance, a recent study showed that curcumin had anti-diabetic effects as it could help to regenerate β-cells in vivo by the immunomodulatory effect on T helper1-related cytokines (IL-2) as well as the immunosuppressive action on IFN-γ, IL-6, and IL-1β [37]. Other study showed that intraperitoneal injections of turmeric (*Curcuma lo*nga) extract, which is the main source of curcumin, could significantly reduce blood glucose level from around 400–100 mg/dL and showed protective effects on pancreatic and renal structure and function [38]. In addition to that, extract of *Sargassum oligocystum* algae, which is rich in phytochemical compounds such as tannins, alkaloids, saponins, and flavonoids, could regenerate β-cells of diabetic rats as well as maintain their function as measured by calculating the homeostasis model assessment of β-cells (HOMA-B) [39].

## 4 Conclusions

According to the results of the in vivo study, WSP exhibited antidiabetic, anti-inflammatory, and antioxidative effects on diabetic rats. Rats given WSP 1.5 and WSP 4.5 had blood glucose, insulin, IL-6, TNF-α, IL-8, and MDA levels as well as SOD activity that were close to the PC group and the WSP treatment showed a dose- and time-dependence during the normalization process. The higher the WSP dose given to diabetic rats, the lower the blood glucose, IL-6, TNF-α, IL-8, and MDA levels and the higher the SOD activity and the insulin level of diabetic rats. Similarly, the longer the WSP intervention in diabetic rats, the lower their blood glucose, IL-6, TNF-α, IL-8, and MDA levels and the higher the SOD activity and the insulin level of diabetic rats. In addition, WSP treatments could significantly improve β-cell regeneration as observed by H&E staining in the end of the study. Taken together, white saffron is a promising aromatic spice having potential antidiabetic effects.

## CRediT authorship contribution statement

**Dwiyati Pujimulyani:** Conceptualization, Methodology, Validation, Resources, Data curation, Writing – original draft, Writing – review & editing, Supervision, Project administration, Funding acquisition. **Wisnu Afi Yulianto:** Conceptualization, Methodology, Validation, Writing – review & editing, Supervision. **Astuti Setyowati:** Conceptualization, Methodology, Writing – review & editing. **Prastyo Prastyo:** Formal analysis, Investigation, Data curation, Writing – original draft. **Sulkhan Windrayahya:** Software, Data curation, Writing – original draft, Writing – review & editing, Visualization. **Ali Maruf:** Software, Data curation, Writing – original draft, Writing – review & editing, Visualization

## Declaration of Competing Interest

The authors declare that they have no known competing financial interests or personal relationships that could have appeared to influence the work reported in this paper.
